# Head Lice in Norwegian Households: Actions Taken, Costs and Knowledge

**DOI:** 10.1371/journal.pone.0032686

**Published:** 2012-02-29

**Authors:** Bjørn Arne Rukke, Tone Birkemoe, Arnulf Soleng, Heidi Heggen Lindstedt, Preben Ottesen

**Affiliations:** 1 Department of Pest Control, Norwegian Institute of Public Health, Oslo, Norway; 2 Department of Ecology and Natural Resource Management, Norwegian University of Life Sciences, Ås, Norway; James Cook University, Australia

## Abstract

**Introduction:**

Head lice infestations cause distress in many families. A well-founded strategy to reduce head lice prevalence must shorten the infectious period of individual hosts. To develop such a strategy, information about the actions taken (inspection, treatment and informing others about own infestations), level of knowledge and costs is needed. The present study is the first to consider all these elements combined.

**Materials and Methods:**

A questionnaire was answered by 6203 households from five geographically separated municipalities in Norway.

**Results:**

94% of the households treated members with pediculicides when head lice were discovered. Nearly half of the households checked biannually or not at all. Previous occurrence of head lice and multiple children in a household improved both checking frequency and method. More than 90% of the households informed close contacts about their own pediculosis. Direct costs of pediculosis were low (less than €6.25 yearly) for 70% of the households, but the ability to pay for pediculicides decreased with the number of head lice infestations experienced. One in three households kept children from school because of pediculosis. Other widespread misconceptions, such as that excessive cleaning is necessary to fight head lice, may also add unnecessary burden to households. School affiliation had a significant effect on checking frequency and method, knowledge and willingness to inform others about own pediculosis.

**Conclusions:**

Increased checking frequencies appear to be the most important element to reduce head lice prevalence in Norway and should be a primary focus of future strategies. National campaigns directed through schools to individual households, might be an important tool to achieve this goal. In addition to improving actions taken, such campaigns should also provide accurate information to reduce costs and enhance the level of knowledge about head lice in households.

## Introduction

Head lice (*Pediculus capitis* De Geer) is an obligate human ectoparasite that is considered a common community health problem [1], [2]. It causes physically uncomfortable pruritus [3] and emotional, economic and social problems in many families [4], [5], [6]. Head lice prevalence varies around the world [7]–[9].

The spread of a directly transmitted parasite in a population of hosts depends on the average time a host remain infectious, the number of susceptible individuals in the host population and the strength of transmission [10]. In the case of head lice, duration of individual infestation (i.e. the time a host is ‘infectious’) depends on inspection (checking method and frequency) and treatment. Pediculicide efficacy has been much studied [11], [12], whereas far less information is available on how inspections are executed. Individual host susceptibility to head lice and transmission rates are also much neglected topics in head lice epidemiology, and more studies are clearly needed [13]–[15]. However, in several European countries contact rates are most frequent among school children [16], and this fits well with the fact that these children are within the age group having the highest prevalence of head lice [1].

Outbreaks of head lice will be a recurring problem in a community where groups of children with tight social bonds suffer frequent reinfestations [17]. Thus, to efficiently control head lice, contact tracing between children and synchronized treatment are necessary [18]. This can only take place if information about own pediculosis is given to others. The importance of synchronized treatment to eliminate infestations from a group of interacting persons within a reasonable time has also been emphasized through mathematical modeling [15]. A satisfactory level of public knowledge regarding pediculosis is needed as it is in the household that the most effective and pragmatic approaches can be taken to fight head lice [19].

To reduce the burden among households suffering pediculosis, both the direct economic costs of treatment and the indirect costs from lost working hours [20] should be kept as low as possible. Furthermore, unnecessary measures such as extraordinary, thorough cleaning of the houses and the preventive use of pediculicides should be kept at a minimum.

In order to develop a well-founded strategy against head lice, information about the actions taken (inspection, treatment and informing others about own infestation) together with level of knowledge and costs related to head lice infestations, is needed. The present survey brings forward such information from households in several regions of Norway. For the first time all these elements are combined in one study.

## Materials and Methods

### Ethics statement

Both The Data Protection Agency of Norway and The Regional Committees for Medical and Health Research Ethics in Norway approved the ethical aspects of this study. Because the study was considered anonymous as each participating household could not be identified directly or indirectly, these institutions also waived the need for written informed consent from the participants.

### Study area

The study was carried out in five geographically separated Norwegian municipalities in September 2008. A total of 42 elementary schools (1^st^–7^th^ grade), nine from Oslo, 11 from Bergen, nine from Trondheim, five from Bodø and eight from Tromsø, participated in the study. All schools were situated in urban settlement areas and had more than 180 students.

### Sampling process

Each child at a participating school received an envelope from the teacher addressed to the parents/carers. Their household was invited to participate in the survey and received a questionnaire, a lice information brochure, a white plastic lice comb (‘PDC’, KSL Consulting, Denmark) and a small zip lock bag. One questionnaire per household was returned. All questions were answered by predefined categories.

The questionnaire was used to elicit information of number of children (<18 years) and adults in the household, lice-checking frequency and method, preventive use of pediculicides, direct economic costs of pediculosis and concern of cost regarding pediculosis treatment (considering not to treat infestation with pediculicides due to high prices). All households were also asked for information on previous occurrence of head lice – that is, if one or more of the household members had earlier suffered pediculosis. If positive, they gave information about number of such infestations, what type of treatment was used, who they informed about their infestations and whether or not the children had been taken out of school when infested. The households were also asked to check their members with the lice comb included and report the findings of pediculosis. To assess the ability to detect head lice, the households were asked to return found head lice in the zip lock bag. Level of knowledge regarding head lice was assessed asking the households to evaluate 12 true or false statements with the response format of ‘true’, ‘false’ or ‘do not know’.

The data on head lice prevalence gathered in this survey is reported by Rukke et al. [9].

### Statistical analyses

Multivariate, mixed-effect (multilevel) logistic regression models were used to analyze the effect several predictor variables had on different binary response variables (checking frequency, checking thoroughness, preventive pediculicide use, informing about own pediculosis, costs regarding treatment of pediculosis and retainment of children from school). Such mixed models contain both fixed effects and random effects, the latter which account for a hierarchical structure of data. In the present study, school was included as a random-effect variable in all models, to account for the fact that study units from the same subpopulations or school could be more similar than those from other schools.

In the analyses checking frequency was categorized as infrequent (less than monthly, only biannually or never) or frequent (monthly or more often), and checking method was categorized as thorough (using lice comb or lice comb and fingers) or not thorough (using fingers, ordinary comb or not checking at all). Because some questionnaires were incomplete, the number of study objects (*n*) differed between the analyses. Statistical analyses were performed using Stata software version 11 [21].

## Results

### Participation

A total of 6203 households submitted the questionnaire. This was the households of one-half of the elementary school students (49.8%, *n* = 16,367) invited to participate in the study. The proportion of participating students varied from 28.5% to 74.9% across schools and from 45.6% to 56.3% across municipalities.

### Actions taken against pediculosis

#### Lice-checking frequencies and methods

**Figure 1:**
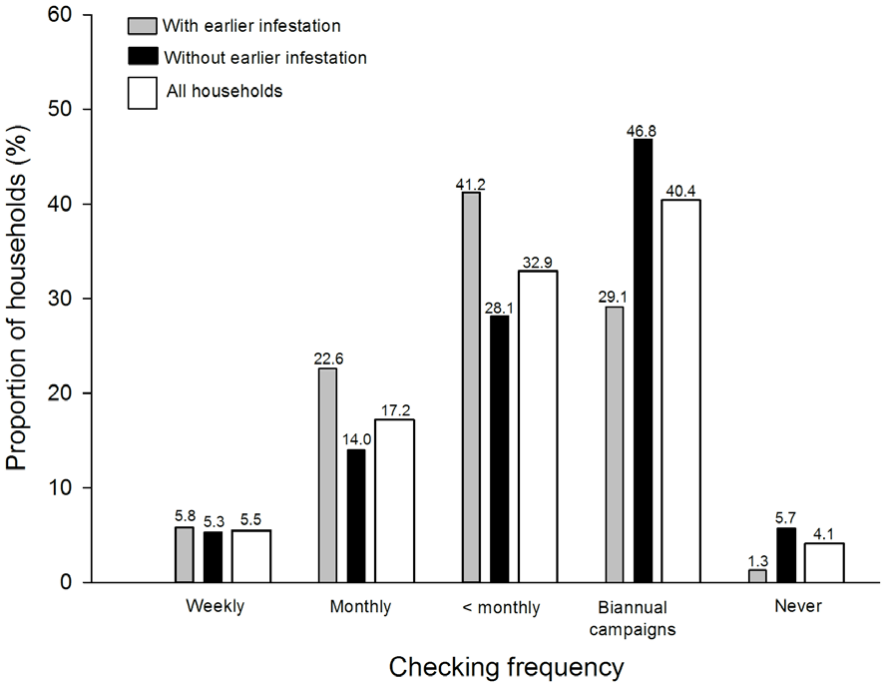
Head lice-checking frequencies. Checking frequencies in households with and without earlier head lice infestations as well as in all households combined. The exact proportion is written above each bar; *n* = 5791.

**Figure 2:**
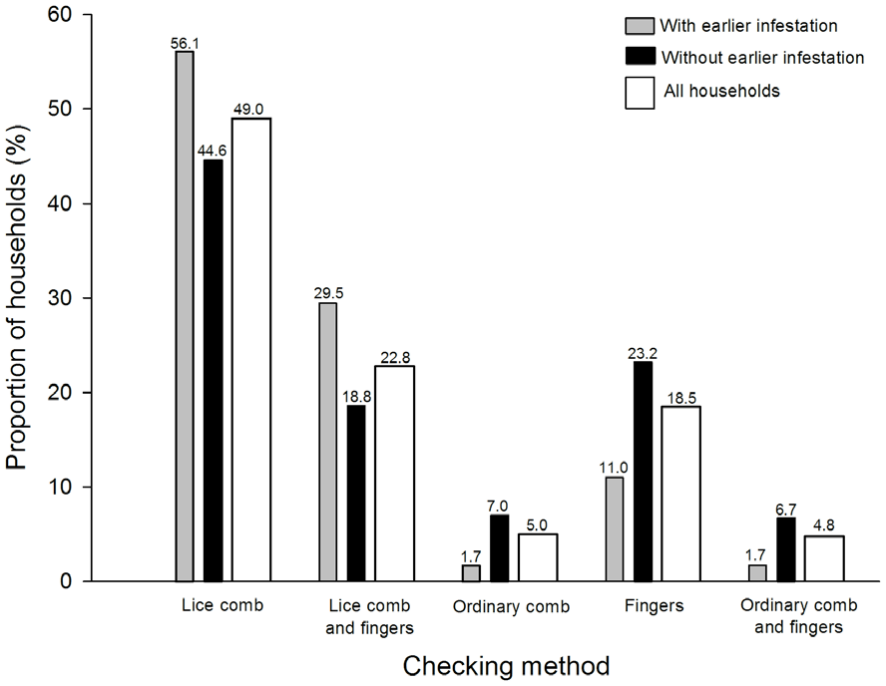
Checking methods for head lice. Checking methods in households with and without earlier head lice infestations as well as in all households combined. The exact proportion is written above each bar; *n* = 5418.

Most households rarely (less than monthly, only in biannual campaigns or never) checked their members for head lice (all households in Figure 1). 40.4% only checked during campaigns, and 4.1% did not check at all. Of those that checked, the majority of households used a lice comb alone or in combination with fingers (all households in Figure 2), while the rest used fingers and/or an ordinary comb when searching for lice. To explain how different household characteristics influenced checking frequency and method, two multivariate models (Table 1 and 2 were created. School, the random-effect variable, significantly improved both models (estimate for frequency: 0.199, *p*<0.001 and estimate for method: 0.241, *p*<0.001). Households that checked frequently for head lice also tended to investigate their members more thoroughly (Tables 1 and 2). Previous occurrence of pediculosis in the household increased both checking frequency and thoroughness (see also Figures 1 and 2). Households in Bergen and Oslo checked more often for head lice than households in other municipalities. With respect to checking method, households in Trondheim were the least thorough. The number of children in the family also influenced checking frequency and method; households with more than three children checked more often than those with fewer children, and households with two or three children checked more thoroughly than households with one child only.

**Table 1:** Model of checking frequency.

Variable	*p* value	Category	Checking often (*n*)	Odds ratio (95% CI)
Number of children	<0.001	1 child	21.1% (1020)	1
(persons <18 years)		2 children	22.0% (3102)	0.99 (0.83–1.19)
		3 children	22.7% (1377)	0.99 (0.80–1.22)
		>3 children	34.6% (292)	1.71 (1.27–2.30)
Number of adults	0.961	1 adult	21.3% (821)	1
(persons >18 years)		>1 adult	22.8% (4970)	1.00 (0.83–1.22)
Municipality	<0.001	Tromsø	15.8% (936)	1
		Bodø	18.6% (575)	1.28 (0.90–1.83)
		Trondheim	19.2% (1398)	1.28 (0.95–1.71)
		Bergen	26.8% (1409)	1.83 (1.40–2.43)
		Oslo	27.8% (1473)	1.91 (1.43–2.54)
Previous occurrence	<0.001	No	19.3% (3675)	1
of head lice		Yes	28.5% (2116)	1.47 (1.28–1.68)
Checking	0.022	Not thorough	19.2% (1901)	1
thoroughness		Thorough	24.3% (3890)	1.18 (1.02–1.37)

Multivariate, mixed-effect logistic regression model of checking frequency (rare or often) in households with school as a random-effect variable. Odds ratios are in relation to the first category of each variable. *n* = 5791.

**Table 2:** Model of checking thoroughness.

Variable	*p* value	Category	Checking thoroughly (*n*)	Odds ratio (95% CI)
Number of children	0.036	1 child	61.0% (1020)	1
(persons <18 years)		2 children	67.6% (3102)	1.24 (1.06–1.45)
		3 children	70.6% (1377)	1.25 (1.03–1.51)
		>3 children	67.8% (292)	1.03 (0.76–1.38)
Number of adults	0.170	1 adult	63.7% (821)	1
(persons >18 years)		>1 adult	67.8% (4970)	1.13 (0.95–1.34)
Municipality	0.001	Tromsø	72.4% (936)	1
		Bodø	66.3% (575)	0.77 (0.54–1.10)
		Trondheim	58.2% (1398)	0.47 (0.35–0.64)
		Bergen	69.7% (1409)	0.66 (0.49–0.88)
		Oslo	70.3% (1473)	0.70 (0.52–0.95)
Previous occurrence	<0.001	No	57.4% (3675)	1
of head lice		Yes	84.1% (2116)	3.88 (3.37–4.45)
Checking	0.027	Rare	65.7% (4481)	1
frequency		Often	72.1% (1310)	1.18 (1.02–1.36)

Multivariate, mixed-effect logistic regression model of checking thoroughness (not thorough or thorough) in households with school as a random-effect variable. Odds ratios are in relation to the first category of each variable. *n* = 5791.

#### Pediculosis treatment

93.9% (*n* = 2168) of the respondents had used pediculicides to treat pediculosis, 71.7% in conjunction with use of lice comb. 5.8% used lice comb as the sole method, and 2.8% reported head shaving as treatment. Only 0.1% of the households took no action when suffering from pediculosis. Malathion was the most commonly used pediculicide (68.1%, *n* = 2088), whereas permethrin and other pediculicides had been used in 6.3% and 8.0% of the households, respectively. Approximately one in four households (26.5%) did not remember which pediculicides they had used.

#### Head lice prevention with pediculicides

8.2%, (*n* = 5767) had used pediculicides as a preventive measure against head lice infestation. Of these, more than half (54.9%) did so because the siblings had head lice. Other reasons among the households for such treatment were information about pediculosis distributed from school (11.0%), pediculosis among friends (22.0%) and/or pediculosis among fellow school/class children (19.5%). A multivariate model with school as a random-effect variable showed that households with earlier occurrence of head lice were more likely to have used pediculicides preventively than those with no earlier infestation (Table 3). Also, households in Oslo and Bergen had used pediculicides for prevention more frequently than other households (differences Bergen vs. Trondheim (*p* = 0.088) or Bodø (*p* = 0.340) were not significant). The school affiliation did not significantly affect preventive use (estimate: 0.176, *p* = 0.126).

**Table 3:** Model of preventive pediculicide use.

Variable	*p* value	Category	Usedpreventively (*n*)	Odds ratio (95% CI)
Municipality	0.008	Tromsø	5.0% (904)	1
		Bodø	5.4% (551)	1.22 (0.72–2.06)
		Trondheim	6.1% (1371)	1.14 (0.75–1.73)
		Bergen	9.4% (1388)	1.53 (1.03–2.26)
		Oslo	11.1% (1420)	1.92 (1.30–2.84)
Previous occurrence	<0.001	No	3.7% (3492)	1
with head lice		Yes	14.9% (2142)	4.37 (3.51–5.43)

Multivariate, mixed-effect logistic regression model of preventive pediculicides use (not used or used) in households with school as a random-effect variable. Odds ratios are in relation to the first category of each variable. *n* = 5634.

#### Informing about own infestation

93.3% (*n* = 2130) of the households informed others about own head lice infestation. Most informed school (79.7%) and the households of their children's friends (70.0%). In addition, some told personnel responsible for leisure activities (10.5%) or the school nurse (5.7%). Households from different municipalities differed in how they informed others about their pediculosis (Table 4), with Bodø and Oslo as the most and least eager informers, respectively. School affiliation significantly affected willingness to tell others about own pediculosis (estimate: 0.633, *p*<0.001).

**Table 4:** Model of informing others.

Variable	*p* value	Category	Informing (*n*)	Odds ratio (95% CI)
Municipality	0.057	Oslo	90.7% (634)	1
		Bergen	95.1% (646)	2.21 (1.04–4.67)
		Trondheim	95.3% (451)	2.19 (0.98–4.90)
		Bodø	98.4% (128)	7.43 (1.46–37.86)
		Tromsø	94.5% (254)	1.86 (0.77–4.51)

Mixed-effect logistic regression model of informing others about own pediculosis (not informing or informing) in households with school as a random-effect variable. Odds ratios are in relation to the first category of the variable. *n* = 2113.

### Costs

The direct cost of pediculosis (i.e. money spent on lice combs and pediculicides last year) was low among most households. 70.0% (*n* = 4539) spent less than 50 Norwegian crowns (NOK, 1.0 NOK≈€0.125at the time of study), and 1.5% used more than 1000 NOK. 10.4% had spent between 250 and 1000 NOK and 18.1% between 50 and 250 NOK. 6.5% (*n* = 1955) of the households that had experienced an earlier head lice occurrence, found the cost of pediculicides so high that they had not treated or considered not treating their children. The multivariate model showed that households with several episodes of pediculosis or a single parent were more concerned about the costs than others (Table 5). Households with more than three children also found the cost higher compared with those with one child (*p* = 0.069) or two children. The school affiliation did not affect concerns about costs significantly (estimate: 0.052, *p* = 0.481).

**Table 5:** Model of costs.

Variable	*p* value	Category	Considerednot to treat (*n*)	Odds ratio (95% CI)
Number of children	0.160	1 child	6.7% (253)	1
(persons <18 years)		2 children	5.7% (999)	1.00 (0.60–1.79)
		3 children	6.8% (573)	1.29 (0.69–2.43)
		>3 children	10.8% (130)	2.06 (0.94–4.49)
Number of adults	<0.001	1 adult	11.7% (247)	1
(persons >18 years)		>1 adults	5.7% (1708)	0.43 (0.27–0.69)
Municipality	0.773	Tromsø	7.8% (219)	1
		Bodø	7.7% (121)	1.11 (0.47–2.61)
		Trondheim	5.7% (437)	0.81 (0.42–1.55)
		Bergen	6.0% (601)	0.72 (0.39–1.34)
		Oslo	6.9% (577)	0.85 (0.47–1.57)
Occurrences of pediculosis	<0.001	Once	5.0% (1206)	1
		Twice	7.4% (512)	1.50 (0.98–2.30)
		Three times	10.6% (160)	2.21 (1.24–3.95)
		>three times	15.6% (77)	3.65 (1.84–7.22)

Multivariate, mixed-effect logistic regression model of the concern of costs regarding pediculicides (never considered not to treat or considered not to treat) in households with school as a random-effect variable. Odds ratios are in relation to the first category of each variable. *n* = 1955.

About one in three households had kept children from school when experiencing head lice infestation (33.2%, *n* = 2021). The multivariate model that included households having experienced one or more earlier head lice occurrences, showed that households in the Bodø municipality, those with a single parent and those using frequent and thorough (*p* = 0.074) checking methods for head lice were more likely to keep their children from school than other households (Table 6). The school affiliation did not affect the likelihood of keeping a child at home significantly (estimate: 0.063, *p* = 0.417).

**Table 6:** Model of children being kept at home.

Variable	*p* value	Category	Retained childrenfrom school (*n*)	Odds ratio (95% CI)
Number of children	0.399	1 child	33.0% (261)	1
(persons <18 years)		2 children	33.5% (1032)	1.11 (0.82–1.49)
		3 children	31.5% (594)	1.03 (0.74–1.43)
		>3 children	38.8% (134)	1.42 (0.91–2.22)
Number of adults	0.024	1 adult	39.2% (263)	1
(persons >18 years)		>1 adults	32.3% (1758)	0.72 (0.54–0.96)
Municipality	0.035	Tromsø	33.2% (241)	1
		Bodø	45.1% (122)	1.62 (1.03–2.56)
		Trondheim	35.8% (436)	1.13 (0.80–1.59)
		Bergen	32.0% (612)	0.92 (0.66–1.27)
		Oslo	30.2% (610)	0.86 (0.62–1.19)
Checking frequency	<0.001	Rare	30.0% (1450)	1
		Often	41.3% (571)	1.64 (1.34–2.01)
Checking thoroughness	0.074	Not thorough	29.1% (320)	1
		Thorough	34.0% (1701)	1.27 (0.98–1.66)

Multivariate, mixed-effect logistic regression model on how households kept children at home children during pediculosis (have not retained or have retained) with school as a random-effect variable. Odds ratios are in relation to the first category of each variable. *n* = 2021.

### Knowledge

#### Statements regarding head lice

More than half of the households answered incorrectly or responded ‘do not know’ to the statements that head lice can survive several days on clothes or furniture, that some pediculicides kill all eggs, that a home with head lice among its inhabitants must be thoroughly cleaned and that head lice easily spread from pillows, furniture, plush animals and clothes (Table 7). More than 90% of the households correctly answered that head lice crawl from head to head, will survive ordinary shampooing and that untreated persons with head lice may repeatedly infest others. Overall, very few households answered all statements correctly (0.3%, *n* = 5613), but more than two-thirds (69.4%, *n* = 5613) responded correctly to more than half of the statements.

**Table 7:** Statements considered.

Statement	Responses		
	Correct	Wrong	Do not know
1. Head lice can jump (False) (*n* = 6000)	72.1%	19.3%	8.6%
2. Head lice can survive several days on clothes or furniture (False) (*n* = 5968)	47.8%	40.3%	11.9%
3. Head lice crawl from head to head in close contact (True) (*n* = 6020)	96.4%	1.8%	1.8%
4. People getting head lice always start to itch immediately (False) (*n* = 5983)	70.7%	19.9%	9.4%
5. Head lice will survive an ordinary shampooing (True) (*n* = 6026)	90.5%	7.5%	2.0%
6. Some available pediculicides kill all lice eggs (False) (*n* = 5933)	32.9%	36.3%	30.8%
7. Only persons having head lice should be treated with pediculicides (True) (*n* = 5996)	74.1%	20.3%	5.6%
8. The home must be thoroughly cleaned if head lice are found (False) (*n* = 5965)	48.5%	41.6%	9.9%
9. Head lice can spread from pets or farm animals (False) (*n* = 5967)	62.7%	13.1%	24.3%
10. Head lice spread easily from pillows, furniture, plush animals and clothes (False) (*n* = 6005)	41.9%	51.4%	6.7%
11. Treatment with pediculicides must be done twice, 8–10 days apart (True) (*n* = 5979)	72.7%	4.6%	22.6%
12. Persons having head lice and who are not treated may infest others repeatedly (True) (*n* = 6013)	96.0%	1.2%	2.7%

Statements regarding head lice considered by the households. The proportion of correct, wrong and ‘do not know’ responses of each statement is given.

#### Ability to identify head lice

Forty-two of the 133 households that reported head lice infestations, collected and returned what they believed were head lice in the zip lock bag. Thirty-six bags contained head lice, while three contained embryonated eggs. The three remaining bags contained empty lice eggs.

## Discussion

### Infectious period of hosts

Although important for the spread of head lice in a human population, the duration of individual head lice infestations has hardly been investigated. Clearly, the duration depends on how quickly the head lice are discovered and subsequently eliminated. In the present study, almost every household (99.9%) treated the infestation when discovered, which is in contrast to Australian and Nigerian studies in which as many as 14% and 22% of the parents, respectively, did not treat infestations [14], [22]. In Norway, the national treatment recommendation of health authorities at the time of the study (2008) was to use a malathion pediculicide combined with louse combing. This was also by far the most frequently used treatment in the present study, indicating that the recommendations were being followed. Until now malathion pediculicides seem to have been effective in Norway (Rukke et al, unpublished results) despite resistance found in the neighboring countries of Denmark [23] and England [24]. Pediculicides are, when used according to the instructions, generally viewed as the most effective treatment for head lice [2].

Even though a child is treated immediately and appropriately after an infestation is discovered, the child can be a source of lice that can infest many others for a long period if the infestation is not discovered early. Infested persons can be totally asymptomatic or in case of primary infestation not develop the characteristic itching (pruritus) for 4 to 6 weeks after being infested [25]. Therefore, to obtain a satisfactory detection rate and reduce the infectious period, inspections must be carried out using appropriate methods on a regular basis [18], [26]. Inspections should be further intensified during peak incidence seasons like late summer and autumn in Europe [27]. Thorough checking with a lice comb should be the preferred method, as it is more effective than visual inspection with fingers or ordinary comb [28]–[32]. More than three-quarters of the households in the present study checked their members less than monthly, and nearly half of the households only checked biannually (in relation to campaigns) or never. The infrequent checking may be a consequence of low prevalence (around 1%) in Norway [9], making infestation a distant phenomenon. Indeed, households having experienced head lice occurrence earlier, checked more often and more thoroughly than those with no prior occurrence.

Once checking, many households should be able to detect head lice if present as more than two-thirds used a thorough checking method (lice comb). However, there is still room for improvement: for instance, in the Trondheim municipality where checking thoroughness was significantly lower than in the other municipalities.

That households with many children checked both more frequently and more thoroughly than households with few children was encouraging as the former households have a higher risk of pediculosis [9]. Increased awareness in high-risk households is needed to quickly discover and treat head lice infestation among their members.

### Informing others and synchronized treatment

It is important to rapidly inform others (e.g. school, parents of playmates and fellow students) when head lice are detected [2], [15], [33] to enable synchronized screening and treatment among acquaintances. Openness can be difficult because of fear of being socially stigmatized [34]. However, this appeared to be a lesser problem among most households in the present study as more than 90% of households informed others about their infestation.

That as much as 40% of the households only checked for head lice during checking campaigns indicate that such campaigns are important fighting pediculosis. The Bug Busting program in the UK is a good example of the positive impact of national campaigns [17]. In Norway in the period 2006–2009 national lice-checking campaigns were launched twice a year by a voluntary organization, ‘Lusfri Norge’. In the years 2007 to 2009 the sale of pediculicides (malathion and permethrin) was reduced by 28%, but sales increased by 9% in 2010 [35] after the national campaigns had stopped. The sale of pediculicides may indicate a possible positive, large-scale effect of campaigns on head lice prevalence in Norway, but this must be confirmed in future studies.

### Preventive use of pediculicides

In an Australian study around 15% of households had used pediculicides for prevention purposes [18]. Even though pediculicides are regarded as relatively safe if used according to instructions, preventive use without identifying the presence of head lice should be avoided [36]. This is also the view advocated by health authorities in Norway. Therefore, it was encouraging that no more than 8% of the households in this study had used pediculicides as a preventive action. Somewhat understandably, more than half of these households chose treatment because other household members were infested. Households which had earlier experienced pediculosis were more likely to have used pediculicides preventively than households with no such experience. Whether or not this was due to other members having head lice at the same time or a heightened determination to avoid new episodes of infestation is not known.

### Economical consequences

Guidelines for head lice control are of little use if households are unable to afford treatment. Expense of commercial treatment products may be a constraint for some families in industrialized countries [37]. In the present study, few households considered not treating head lice for financial reasons. However, among those that had suffered pediculosis repeatedly cost was clearly an issue as the willingness or ability to pay for treatment decreased with number of head lice infestations. The same applied to households with one adult or many children. This suggests that economic compensation, like in UK where all persons have the right to free head lice treatment [38], could be considered at least for certain households.

Indirect costs of lost working hours for parents and school time for children seem to be a considerably larger problem than the direct costs. As many as one in three households had kept infested children away school for one day or more when experiencing head lice. In an Australian study, 24% and 30% of all households in two different regions had kept their children from school because of head lice [18]. Counting all households in the present study the comparable figure is 11%. A ‘no nit’ school attendance policy is an impractical biological measure for preventing further infestations [5] and, once treated, children should not have to stay home from school and miss educational opportunities [39]. This has been an important element in the head lice guidelines given by Norwegian health authorities.

Households with higher checking frequency and thoroughness kept children at home more than others. Apparently, taking head lice more seriously also includes missing more days at work because of pediculosis. Single parents were also more likely to keep their children at home. Assuming that these individuals have a larger number of days off work because of illness in the children than other parents, head lice infestations will add a larger burden to single parents than to two-parent households.

### Knowledge

That two-thirds of the households answered at least half of the statements correctly is comparable with the level of parental knowledge found in Australia [18]. Both studies included similar statements and distributed a head lice information pamphlet to the parents together with the questionnaire.

The present study revealed important knowledge gaps. More than half of the households erroneously thought that some pediculicides kill all eggs (false at the time of questioning), thorough house cleaning is necessary to fight pediculosis, head lice survive long periods away from a scalp and spread easily through fomites. Unnecessary, thorough cleaning of bedding, clothes, soft toys and the house environment [14] can be a heavy burden for a family [37], and may take the focus away from more important actions as treatment of scalps, inspection of other family members and informing social contacts about own infestation.

The ability of parents, health care providers and others to identify head lice has shown to be poor in some studies [36], [40]. In the present study, however, the ability to recognize an infestation seemed better, as nearly all households returning a zip lock bag identified head lice correctly. One may argue that as only 30% of the households with pediculosis returned the plastic bag, the data may not representative. This is difficult to evaluate and should be investigated in future studies.

### Importance of municipality and schools

Schools impart knowledge and create attitudes among students and their households. A range of treatment and monitoring approaches can be applied by different schools [37]. In the present study, school affiliation seemed important for checking frequency and method as well as willingness to inform others about own pediculosis. These elements are important to counteract head lice infestations, and information given out by schools regarding this should be correct. The differences found indicate that there is room for improvement in some schools. Preventive use of pediculicides and the decision to keep infested children away from school were not affected by school affiliation. Thus, whether or not a school distributes national recommendations, these choices seem likely to be taken by the individual households themselves.

At an even larger scale, observed differences between municipalities imply that regional differences exist. Higher prevalence in Oslo and Bergen [9] may have contributed to a larger focus on head lice and explain why households in these municipalities checked their members and used pediculicides more preventively than households from other municipalities. Regarding information given about own head lice infestations, this was best in Bodø and poorest in Oslo. It is possible that this contributed to the lower head lice prevalence observed in Bodø compared with Oslo [9]. Furthermore, more households kept students at home during pediculosis in Bodø than elsewhere. Clearly, the observed differences between municipalities imply that regional trends even within a country should be considered when drafting future guidelines, information material and other measures against pediculosis.

### Strategy against head lice

Even though the average head lice prevalence among Norwegian household members was as low as 1%, more than one-third of households had experienced prior occurrences of pediculosis [9]. To reduce this number, the duration of individual infestations should be targeted. In the present study, nearly all households fought head lice when discovered, and the treatment seemed appropriate. Checking frequencies, however, were suboptimal and ought to be improved. As a large proportion of households only inspected members in connection with nationwide biannual campaigns, maintaining these should be considered as they probably are important to obtain synchronized inspection and treatment. At the same time, the need for additional, more frequent inspections should be emphasized. Intensified inspections during peak incidence seasons like late summer and autumn in Europe [27] will also be advantageous.

The present study also revealed important knowledge gaps among households. These ought to be focused on in future campaigns to reduce the work load of parents. For instance, it should be clearly stated that a child should not be kept at home after treatment has started, and that excessive house cleaning following detection of an infestation is unnecessary.

Schools' potential as powerful influencers of modus operandi against pediculosis should be utilized. They can distribute information from health authorities and urge students and their households to participate in head lice campaigns. Schools with high prevalence could also distribute specially targeted information to intensify inspection among households for some time. Also, as the number of head lice episodes increases most during the first years of elementary school [9], comprehensive information for the households of first-grade students should be considered.

Finally, steps to ensure that lower-income households are supported financially to fight head lice might be considered by authorities.

### Limitations of the study

Households of about half of the invited students participated in the present study. This is similar, or slightly better, than other head lice studies based on caretaker feedback [18], [19], [41]. However, we can not rule out that a selection bias may have affected the results (e.g. [42]). Being a retrospective investigation, the present study has inherent uncertainty due to the time lag between head lice infestations and reported information. However, the large sample size in our study reduces the possibility that some erroneous answers will incorrectly influence the general conclusion.

### Conclusions

The present study shows that treatment of head lice infestations, once it was detected, was satisfactory in households of elementary school children in Norway. Also, most households informed about own infestation enabling others to check household members when head lice had been in detected in the local environment. The main challenge appears to be reduction of the infectious period prior to detection, a period when head lice can spread unnoticed from head to head. Checking method was comprehensive, but checking frequency ought to be improved to achieve this goal.

The direct costs of pediculosis were low, even though repeated infestation influenced the ability to treat in some households. The indirect costs appear higher as one in three kept their children away from school during infestation giving lost school and working hours in households. Also, other misconceptions, like the need for thorough house cleaning during head lice infestation, were widespread.

To counteract the negative consequences of pediculosis, head lice campaigns directed through schools are likely to be an efficient tool to improve actions taken, reduce costs and increase knowledge regarding such infestations.
